# Search of low-contrast liver lesions in abdominal CT: the importance of scrolling behavior

**DOI:** 10.1117/1.JMI.7.4.045501

**Published:** 2020-07-24

**Authors:** Alexandre Ba, Marwa Shams, Sabine Schmidt, Miguel P. Eckstein, Francis R. Verdun, François O. Bochud

**Affiliations:** a Lausanne University Hospital and University of Lausanne, Institute of Radiation Physics, Lausanne, Switzerland; b University of Lausanne, Lausanne, Switzerland; c Lausanne University Hospital and University of Lausanne, Department of Radiology, Lausanne, Switzerland; d University of California Santa Barbara, Department of Psychological and Brain Sciences, Santa Barbara, California, United States; e University of California Santa Barbara, Department of Electrical and Computing Engineering, Santa Barbara, California, United States

**Keywords:** visual search, scrolling behavior, image perception, observers’ performance, computed tomography, radiologists’ strategies

## Abstract

**Purpose:** Visual search using volumetric images is becoming the standard in medical imaging. However, we do not fully understand how eye movement strategies mediate diagnostic performance. A recent study on computed tomography (CT) images showed that the search strategies of radiologists could be classified based on saccade amplitudes and cross-quadrant eye movements [eye movement index (EMI)] into two categories: drillers and scanners.

**Approach:** We investigate how the number of times a radiologist scrolls in a given direction during analysis of the images (number of courses) could add a supplementary variable to use to characterize search strategies. We used a set of 15 normal liver CT images in which we inserted 1 to 5 hypodense metastases of two different signal contrast amplitudes. Twenty radiologists were asked to search for the metastases while their eye-gaze was recorded by an eye-tracker device (EyeLink1000, SR Research Ltd., Mississauga, Ontario, Canada).

**Results:** We found that categorizing radiologists based on the number of courses (rather than EMI) could better predict differences in decision times, percentage of image covered, and search error rates. Radiologists with a larger number of courses covered more volume in more time, found more metastases, and made fewer search errors than those with a lower number of courses. Our results suggest that the traditional definition of drillers and scanners could be expanded to include scrolling behavior. Drillers could be defined as scrolling back and forth through the image stack, each time exploring a different area on each image (low EMI and high number of courses). Scanners could be defined as scrolling progressively through the stack of images and focusing on different areas within each image slice (high EMI and low number of courses).

**Conclusions:** Together, our results further enhance the understanding of how radiologists investigate three-dimensional volumes and may improve how to teach effective reading strategies to radiology residents.

## 1 Introduction

Colorectal cancer mortality in Europe affects 190,000 patients per year, and it is estimated that 50% of patients die from hepatic metastases.^1^ Hepatic metastases are already present when the cancer is diagnosed in 30% to 40% of cases,^2^ and the only known curative treatment is the resection of the primitive tumor together with metastatic disease.^3^ This means that rapid and effective detection of liver metastases is essential to improve prognosis.^1^

Various volumetric imaging modalities can be employed to detect and characterize hepatic metastases; the most commonly employed is helical computed tomography (CT).^2^^–^^4^ Its sensitivity mainly depends on technical factors, such as image acquisition and reconstruction parameters but also on the features of the detected metastasis, such as size and contrast^2^ and reader’s capacity.

To maximize metastases detection, the contrast between the hepatic parenchyma and the metastases is augmented using an intravenously injected contrast agent.^2^ During the venous phase, the latter typically appears as hypodense lesions surrounded by the contrast-enhanced homogeneous liver parenchyma, increasing the sensitivity of detection to 80% on average.^2^ Nonetheless, the way in which radiologists search through the high number of axial CT images can also affect the effectiveness of detecting the metastases, and strategies can substantially vary between radiologists.^5^^,^^6^

Image perception studies play an important role in understanding the radiologists’ perceptual and cognitive processing of medical images.^7^^,^^8^ Characterizing how radiologists explore medical images may help to improve the detection of hepatic metastases. For this purpose, eye-tracking studies have been used to gain insight into the radiologist’s ability to search and recognize various targets^5^ in different imaging modalities.^9^

A recent study on chest CT^6^ showed that radiologists tend to follow two main reading strategies as they scan or drill through multislice CT images. According to Drew et al.,^6^ drillers focus on a small part of the organ while quickly scrolling images forward and backward, and scanners scan each level of the entire organ before moving to the next slice and thus advance more slowly but investigate a larger area. They found that drillers are more efficient in performing a visual search task, finding more lesions, and covering more lung volume on average. The study categorized readers as drillers or scanners based on an eye movement index (EMI) that quantifies the tendency of radiologists to make large saccades. However, the EMI does not consider how readers scroll through the different slices in the volumetric data. A radiologist might execute small saccades (low EMI) but still scroll through a small fraction of the slices. On the other hand, the reader might execute large saccades and scroll through most slices. Thus, scrolling behavior might contribute to the search performance independently from saccade amplitudes. However, few studies have been reported on eye-tracking experiments coupled with scrolling in volumetric images. Our first goal was therefore to develop more comprehensive metrics of eye movement search patterns with three-dimensional (3-D) volumes that would include the number of scrolls between fixations.

Although the effect of signal detectability on performance^10^^–^^12^ and eye movements^11^^–^^13^ has been examined with search in two-dimensional (2-D) displays, little is known about its influence on search with 3-D volumetric data. Indeed, most eye-tracking studies in volumetric images have focused on a single type of target without considering the possible influence of signal features (signal size, shape, or contrast) on search effectiveness and strategies.^6^^,^^9^^,^^10^ Our second goal was to evaluate the effect of signal contrast on 3-D search patterns for high and low contrast targets in volumetric CT images.

We designed a psychophysical experiment that tracked radiologist eye position and classified fixations and saccades in multiple CT slices, coupled with a measure of scrolling behavior. Twenty radiologists with variable training experience participated in the study. We instructed them to perform a free search task of lesions with two low-contrast levels to estimate their diagnostic performance and to identify eye movement and scrolling patterns that characterize search in volumetric images.

## 2 Materials and Methods

### 2.1 Liver CT Data

#### 2.1.1 CT acquisition

Our retrospective collection of patient examinations was approved by the local ethics board (protocol number: 466/14). We included 15 anonymized intravenously contrast-enhanced abdominal CT examinations from our hospital’s database. In all cases, the liver parenchyma had been reported as normal, in particular without any focal lesion nor diffuse steatosis. The examinations were performed on a 64-detector row CT machine (Discovery 750HD, GE Healthcare; Milwaukee, WI, USA). We performed a routine abdominal acquisition following our standard clinical oncological protocol [120 kV, 300 to 400 mA, table speed 55 mm rotation (0.6 s), pitch 1.275, and axial slice thickness/reconstruction interval 2.5  mm/2  mm]. CT images were reconstructed according to our routine default setting, including filtered back projection and adaptive statistical iterative reconstruction with 25% blending. We intravenously injected iodinated contrast medium (Accupaque^®^, Iohexol, 300  mgI/ml, GE Healthcare, volume in milliliters=body weight+30  ml) at a flow rate of 3  ml/s. We used automatic tube current modulation in all 3 axes (SmartmA).

#### 2.1.2 Cases preparation for reader study

Stimulus material used for the reader study was hybrid CT images generated by inserting a synthetic low-contrast volumetric signal mimicking a hypodense focal liver lesion. The signal size was 8 mm, which subtended a 0.8-deg visual angle on the reader’s eye for the experiment setting. The signal profiles in all directions were fitted to real liver lesion profiles. We used the alpha blending technique that removes anatomical structures from the volume of interest and replaces it with another obtained by blending a uniform region and the signal.^13^ An experienced radiologist designated the locations in the liver parenchyma free of main structures (veins and arteries) for signal insertion. Two sets of 15 distinct cases were created by inserting one to five low contrast signals (average=3  signals) in each case. The first set contained 49 signals with contrast of −50 Hounsfield units (HU). The second set contained 45 signals with contrast of −30  HU. There were no cases with no signals. The resulting sets of hybrid images were visually assessed by an experienced radiologist. Each case was composed of 100 consecutive slices containing the whole liver.

### 2.2 Reader Study

To track and record the reader’s gaze, an eye-tracking device (EyeLink1000, SR Research Ltd., Mississauga, Ontario, Canada) was positioned below the image display and calibrated to maintain the average gaze error below 1 deg. Fixations were detected using the default parameters: eye velocity and acceleration thresholds of 30  deg/s and $9500\;\deg/s^{2}$, respectively. The participants were seated in front of a 22 in. (56 cm) screen suited for medical image display in a reading room with low illuminance (<50  lux). The participant’s head position was fixed with a forehead- and chin-rest mount to improve accuracy in eye gaze measurements and control the visual angles. Before each reading session, a calibration procedure was applied to ensure a good eye-tracking accuracy. An additional eye-tracking drift check was performed between each trial. The cases were presented with a magnification factor of 2 with a window level of 50 HU and a width of 300 HU. Readers had no possibility to zoom or pan the images, neither to adjust the image contrast.

Using a mouse wheel, the readers could freely scroll forward and backward through all the slices and were instructed to mark a lesion with a mouse click. Before the actual trials, they were shown examples of the signal to be searched, and they were informed that each case contained at least one lesion to localize. No time limitation was imposed to encourage a thorough evaluation of each case.

In total, 20 readers took part in the experiment with reading expertise ranging from 1 to 17 years. In terms of demography, the reader group consisted of one undergraduate medical student, sixteen 1 to 5 year radiology residents, 3 fellows with 5 to 8 years of clinical experience in body imaging, and one radiologist with an experience of 17 years in abdominal CT imaging. However, for one participant (5 to 8 years of clinical experience in body imaging group), low calibration accuracy resulted in unreliable eye position data. These participant data were therefore removed from our study.

### 2.3 Data Recording and Analysis

From the first scrolling wheel activation until the end of the trial, the eye gaze position in x, y (within slice coordinates), and z (slice number) was recorded at 60-Hz rate. The marker position was recorded when the readers localized a lesion.

From the raw gaze data and marker positions, we derived the following measurements: localization hit rate, perceptual and search error rate, search duration, saccades amplitude, liver coverage, and strategy quantification. A reader’s marking was considered a localization hit when it fell into a disk centered on the lesion’s “center of mass,” whose radius was twice the radius of the lesion. The hit rate was defined as the number of lesions correctly marked compared with the total number of lesions. A perceptual error refers to a missed lesion that was fixated.^14^^,^^15^ A lesion was considered fixated if it was encompassed by a 2–deg-diameter circle centered on the recorded fixation locations. A search error refers to a missed lesion that was not fixated (encompassed by 2-deg–diameter circles around the fixations). The search time was considered the time measured between the first fixation onto the liver parenchyma and the moment the reader decided to terminate the trial. The saccade amplitude was defined as the distance between two consecutive fixations, measured in degrees.

The coverage was defined by the liver volume encompassed by a gaze cone defined by a 5-deg diameter disk centered on the gaze coordinate. Every point of the image that fell within the 5-deg gaze cone was considered as visible. We chose 5 deg to be consistent with the literature and the concept of the useful field of view (UFOV).^15^ For a −50-HU signal contrast, more than 70% of the detection saccades were within 5 deg of the previous fixation, and for a −30  HU signal contrast more than 87% of the detection saccades were within 5 deg.

To classify the readers according to their strategy, we measured their EMI.^6^ This parameter had already been developed for the detection of lung nodules,^6^ and we extended it to focal liver lesions.

### 2.4 Search Strategy Metrics

**Fig. 1:**
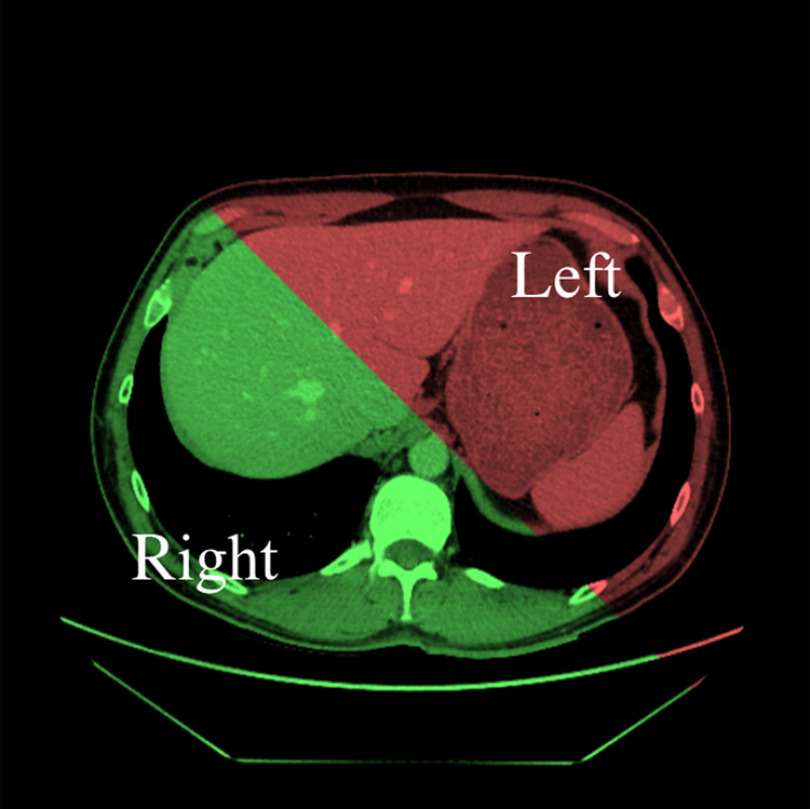
Example of one liver slice from our study with colored overlay showing left and right parts of the liver. On the anatomical level, the left and right liver are defined differently but we chose to separate them according to the left and right parts of the screen. This makes it possible to have two almost similar volumes while anatomically the right liver represents the largest part of the organ. Having two of the same volumes is essential for the detection of crossovers because the number of crossovers is defined as the number of times a saccade crosses the line delimiting left and right liver during a trial. Furthermore, this separation facilitates the division of the organ at each test.

We first followed previous approaches to categorize drillers and scanners using the EMI.^6^ The EMI was derived from the summation of two components: (1) the saccadic amplitude, measured in degrees and (2) the time-averaged number of crossings over a line that delimits the left and right parts of the liver, measured in $s^{-1}$ (Fig. 1). Before doing the summation, both quantities were normalized to the maximum value relative to the readers’ population. The only variation of the definition given by Drew et al.^6^ is that the eye movement crossovers in the lung CT study were defined across quadrants while in the current study we measured crossovers across the left and right part of the liver.

Drew et al.^6^ classified readers based on the EMI into two categories of search strategies: drillers go back and forth across slices during the trial, and each time they tend to fixate on a different area of the image. The few eye movements in the (x,y) plane are compensated by many back and forth scrolls across image slices (z). In contrast, the scanners scroll in one direction throughout the image stack and tend to explore each image slice one after the other through multiple fixations. The use of this search strategy for the scanners results in a high-EMI value.

Because the scanners might also perform fewer back and forth scrolling than the drillers, we decided to measure the number of courses, which we defined as the number of times a reader scrolled in a given direction during the test. For instance, a reader who scrolled through the image stack in one direction, then reversed through a couple of image slices and finally scrolled again in the original direction until the last slice would have performed three courses.

To evaluate a potential relationship between the EMI and the number of courses, we first computed the mean values of both parameters for each reader. We then labeled each reader as having either a high or a low EMI, and, respectively, a high or a low number of courses. The threshold between high and low categories was defined by the median value among all the readers. Therefore, a reader was labeled as a high EMI with a capital “E” if his or her mean EMI was above the median value computed among all the readers. Conversely, a reader with a mean EMI lower than the median was labeled with a lowercase “e.” We did similarly with the number of courses: a reader with a mean number of courses higher than the median of all the readers was labeled with a capital “C,” and a reader below the median was labeled with a lowercase “c.”

Because the images contained multiple lesions, we quantified the hit rate as the number of localized lesions divided by the total number of lesions across all cases (N=49 in −50  HU image set, N=45 in −30-HU image set). Similarly, the miss rate was quantified as the number of missed lesions divided by the total number of all lesions across all cases. We also defined, as search errors, those lesions that were not fixated and missed (not localized).^8^^,^^14^^,^^16^ Perceptual errors were defined as lesions that were fixated and missed.^8^^,^^14^^,^^16^

To assess statistical significance, we used parametric t-tests. Independent samples t-tests were used when comparing separate groups of radiologists classified based on the EMI or number of course criteria. Paired t-tests were used when comparing the same individuals across signal contrast conditions. We used Pearson correlations to quantify the relationship between various variables. The analyses were performed with the Microsoft Excel 2016 Analysis ToolPak.

## 3 Results

### 3.1 Characterization of Reader Strategy and the Role of Scrolling Behavior

**Fig. 2:**
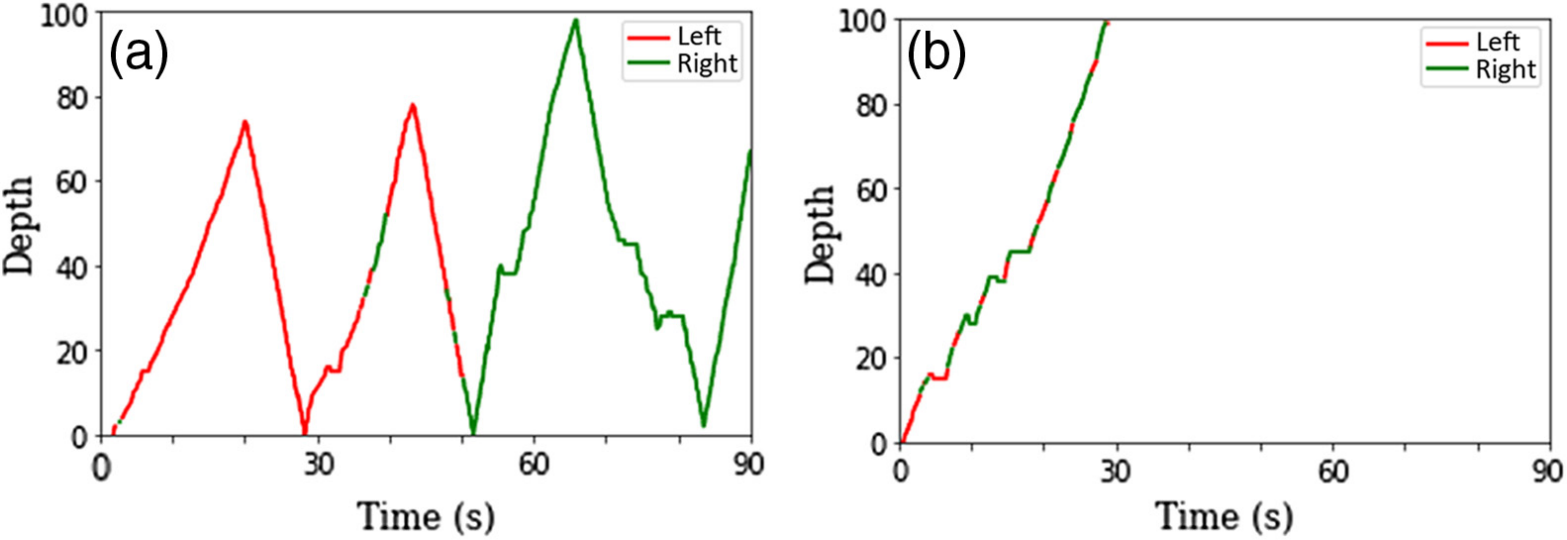
Depth (slice number in the z direction) versus time plot example for typical (a) driller and (b) scanner. In this example, the number of courses per trial was 7 for the driller-like reader and 1 for the scanner-like reader. Colors indicate which part of the liver was analyzed by the reader. For driller, each part was individually fixated while scrolling, and for scanner both parts, left and right liver were alternatively fixated while scrolling.

The number of courses was estimated by plotting the image slices (slice number in the z direction) versus time for each trial and each reader. Figure 2 shows two archetypical examples of scrolling behavior: one with seven courses consistent with driller strategy (left) and another with a single course highly compatible with the behavior of a scanner, as described in Ref. 6.

**Fig. 3:**
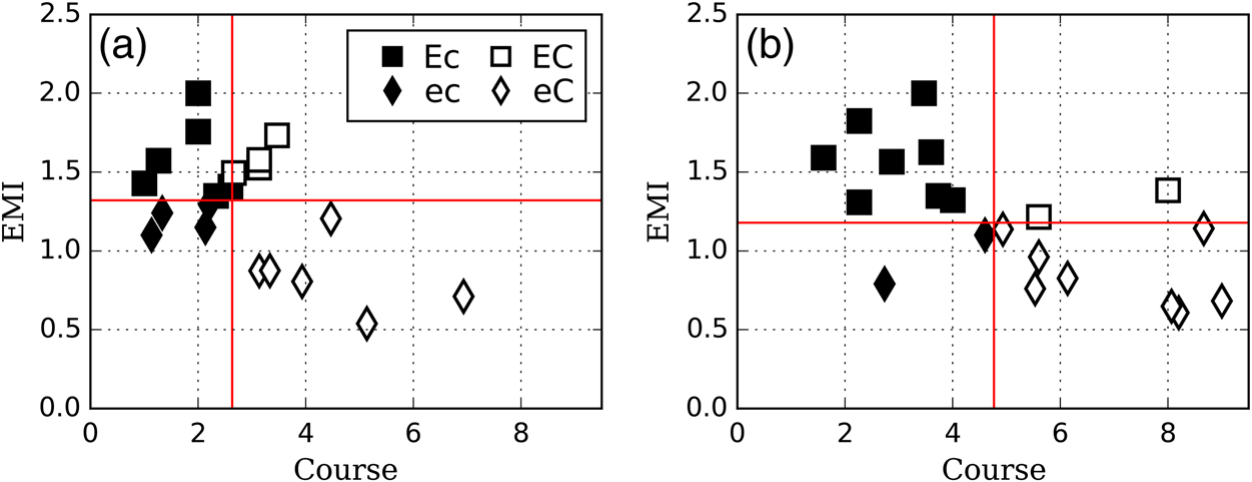
EMI versus the average number of courses over all trials for each signal contrast: (a) −50 and (b) −30  HU. Plain lines correspond to median value of EMI and number of courses across the entire sample of readers. Readers were grouped according to their EMI and number of courses in comparison to median values across the entire sample.

Figure 3 shows the relationship between EMI and the mean number of courses for each of the readers in our study. We classified the readers based on the four reader categories delimited by the medians of the EMI and number of courses parameters (see Sec. 2). The first group is identified as “Ec” for high EMI and low number of courses, the second group is “EC” for high EMI and high number of courses, the third group is “ec” for low EMI and low number of course, and the fourth group is “eC” for low EMI and high number of courses. The most experienced radiologist (17 years) falls into group Ec in both higher and lower contrast case study.

For both signal contrast values, readers with a high EMI tend to have a small number of courses and vice versa. However, while this is significant for the higher contrast (r=−0.56; p=0.01), it is not the case for the lower contrast (r=−0.26; p=0.27).

Comparing the behavior of the individual readers across contrasts (higher contrast versus lower contrast) showed that 7 readers out of 20 changed their EMI category (1 from Ec to ec, 3 to EC to eC, and 3 from eC to EC), and only 1 reader changed the number of courses (from ec to eC). No reader changed both EMI and number of courses. This finding suggests that a categorization of readers based on the number of courses might be more invariant across signal contrast conditions than using the EMI.

**Fig. 4:**
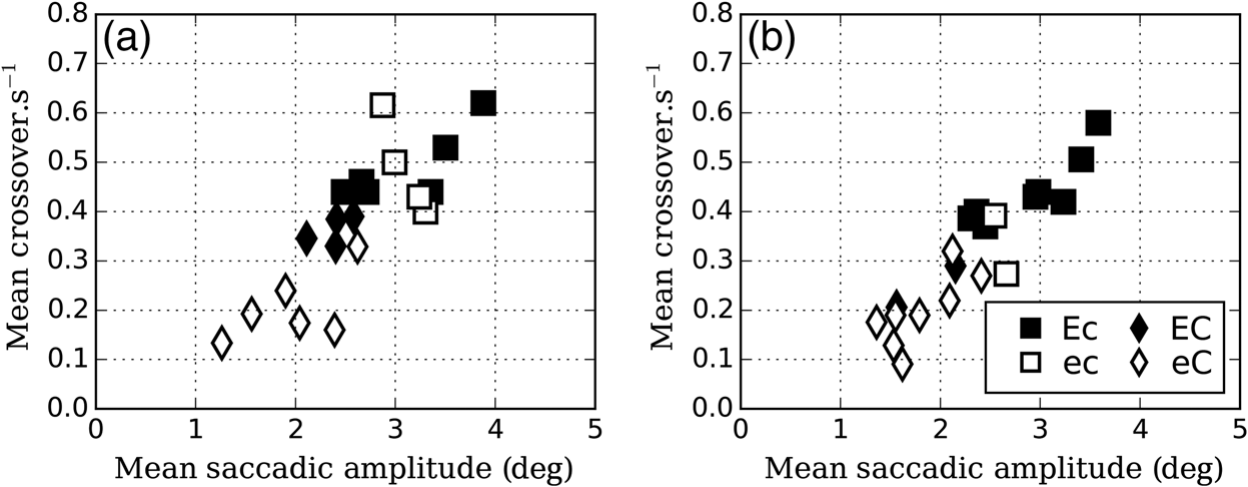
Relationship between the two parameters that define the EMI. Mean crossover per second versus mean saccadic amplitude for (a) −50 and (b) −30  HU.

Figure 4 shows the dependence of the two quantities that define the EMI: the mean crossover per second versus the mean saccadic amplitude. There is a positive correlation between these two quantities, with r=0.82 (p<0.01) and r=0.92 (p<0.01) for −50 and −30  HU, respectively.

The fact that the correlation between the two variables composing the EMI (the crossovers and the saccade amplitude) is higher than the correlation of the EMI and the number of courses suggests that the latter adds additional information to characterize the reader’s search strategy.

### 3.2 Search Performance

**Fig. 5:**
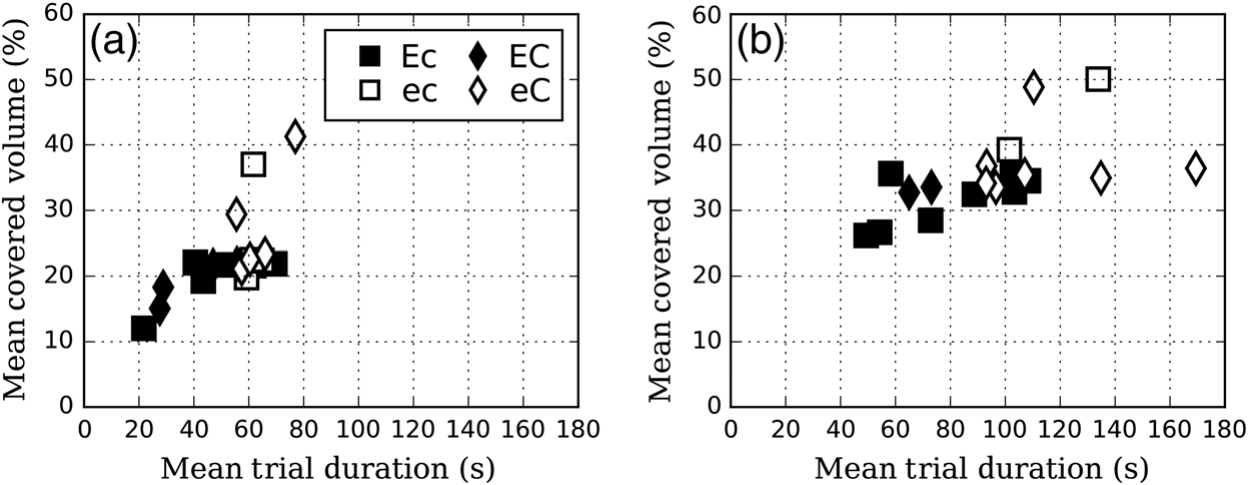
Liver volume coverage with respect to trial duration for (a) −50 and (b) −30  HU.

In this section, we investigated how radiologists categorized along the various search strategy characteristics (EMI and number of courses), differ in basic visual search measures such as the UFOV (mean covered volume) and the trial decision time (mean trial duration). Figure 5 shows the relationship between the liver volume coverage and the mean trial duration. Readers with a high number of courses (eC and EC) tend to cover more volume [at −50  HU (p=0.03) and at −30  HU (p=0.01)] in more time [at −50  HU (p<0.01) and at −30  HU (p<0.01)] than readers with a low number of courses (Ec and ec). No trend was observed in terms of covered volume or trial duration when we look at the readers’ EMI (all p-values >0.4).

The covered volume is positively correlated with trial duration for the high contrast (r=0.68, p<0.01) and for the low-contrast signal (r=0.58, p<0.01). As expected, the decrease in signal contrast tends to increase the need to thoroughly search the volume (the coverage; p<0.01) and the duration of trials (p<0.01).

**Fig. 6:**
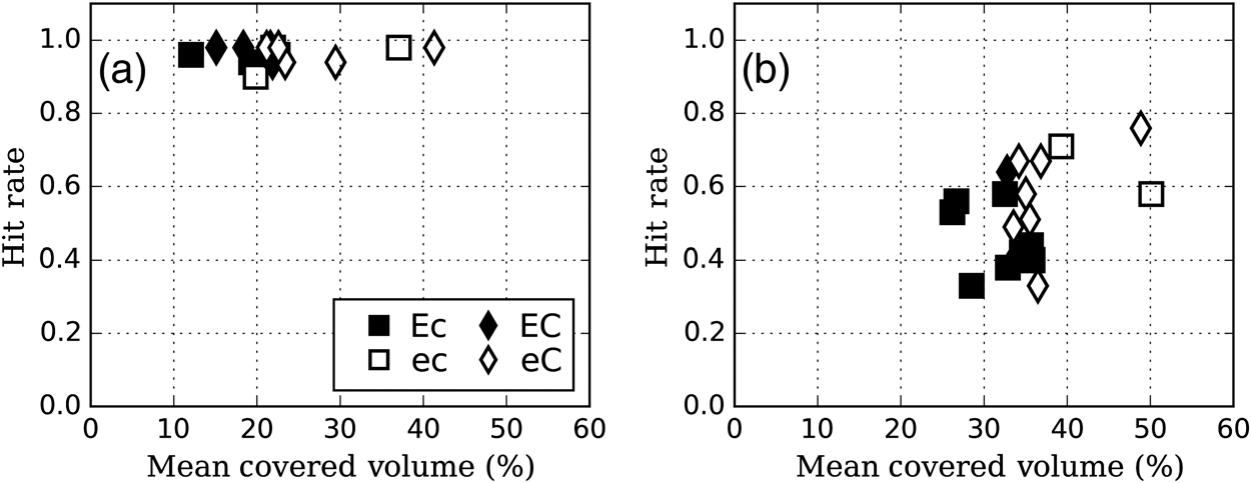
Liver volume coverage with respect to localization hit rate for (a) −50 and (b) −30  HU.

Figure 6 shows the relationship between the localization hit rate and the liver volume coverage. The results show a ceiling effect at the −50  HU signal contrast and thus the hit rate varies with the number of courses or EMI (p=0.6 for the number of courses and p=0.3 for EMI). However, the hit rate at −30  HU signal contrast is significantly higher for readers with higher number of courses (difference in mean hit rate=0.44; p=0.04) than for these with a lower number of courses [Fig. 6(b)]. This was not the case when we categorized the readers on the basis of a high and low EMI (difference in mean EMI=0.1; p=0.3).

**Fig. 7:**
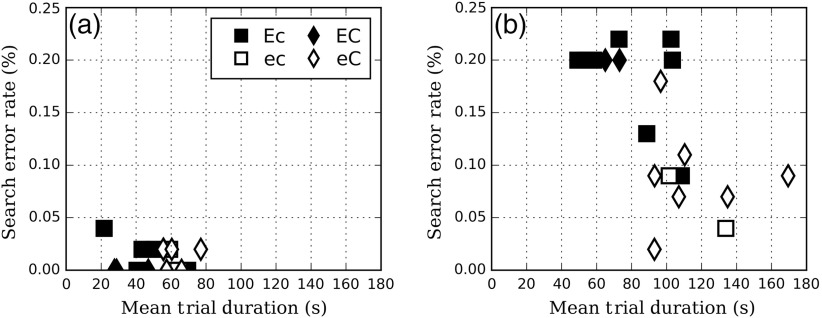
Search error rate versus trial duration for (a) −50 and (b) −30  HU.

Figure 7 shows the search error rate (missed lesion that were not fixated) versus the trial duration. As expected, the −50  HU contrast images [Fig. 7(a)] led to shorter observation times and significantly lower search errors than −30  HU [Fig. 7(b)] for all groups. For the −30  HU signal contrast, readers with a high number of courses (eC and EC) tended to have longer trial durations [mean difference=42  s; p<0.01; CI95% (100,131) compared to CI95% (64,91)] and lower search error rates [mean difference=0.13, p<0.01; CI95% (0.06,0.12) compared to CI95% (0.16,0.21)] than readers with a low number of courses (Ec and EC). However, a change of EMI did not result in a significant difference neither for trial durations [mean difference=18  s; p=0.2; CI95% (78,125) for low e; CI95% (78,104) for E] nor for search error rates [mean difference=0.04; p=0.2; CI95% (0.08,0.18) for low e; CI95% (0.12,0.19) for E].

**Fig. 8:**
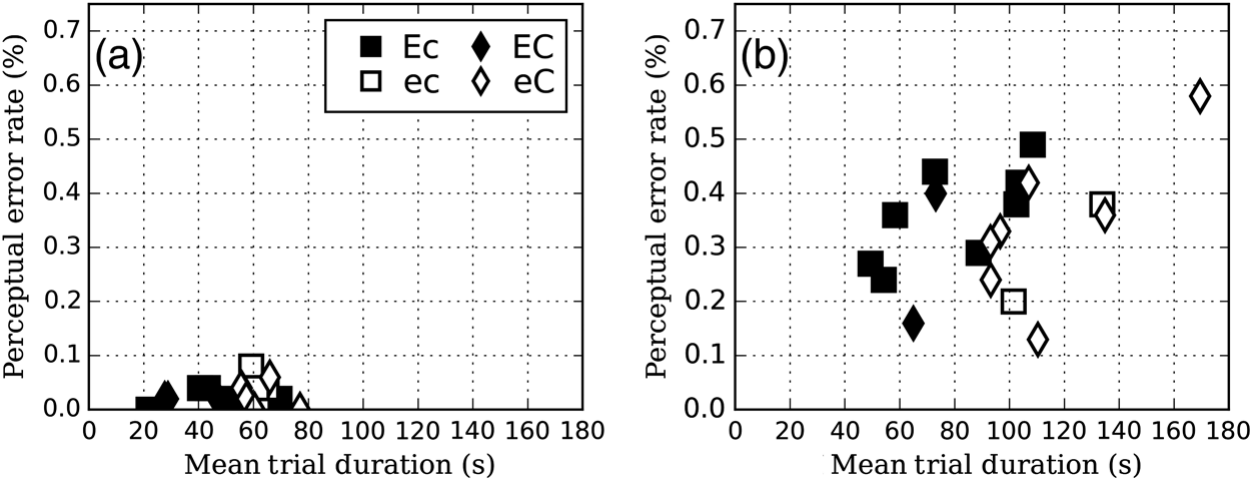
Perceptual error rate versus trial duration for (a) −50 and (b) −30  HU.

Figure 8 shows the perceptual error rate (missed lesions that were fixated) versus the trial duration. The results show that the −50  HU contrast images led to significantly fewer errors than −30  HU contrast images (p<0.01). The perceptual error rate was not dependent on the number of courses (mean difference=0.31 ; p=0.5 for −50  HU and mean difference=0.04, p=0.8 for −30  HU) or the EMI (mean difference=0.01, p=0.3 for -50 HU and mean difference=0.04, p=0.7 for −30  HU).

### 3.3 Effect of Signal Contrast on Search Strategy

**Fig. 9:**
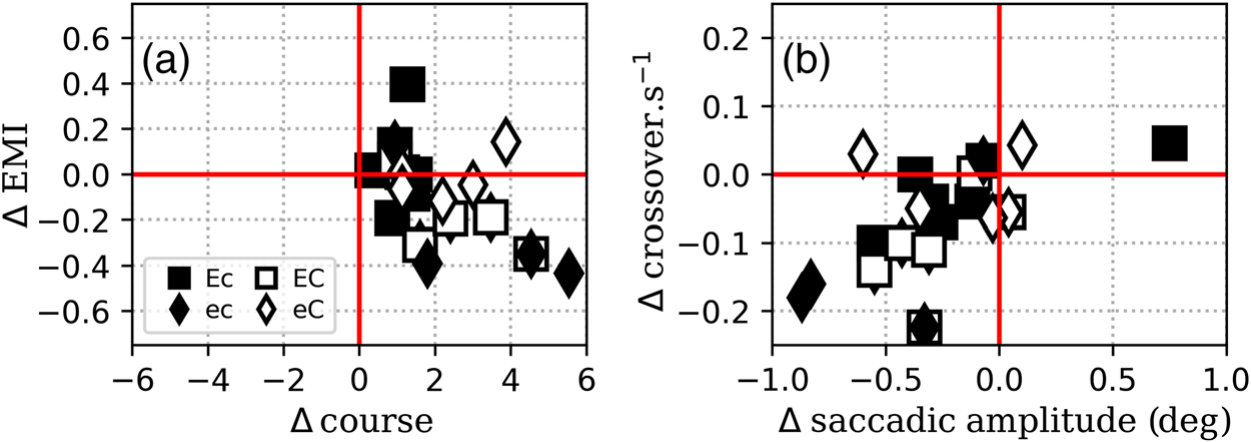
Effect of the signal contrast on EMI and number of courses (Δ is the difference of the considered parameter from −50 to −30  HU). (a) ΔEMI versus Δcourse. (b) Decomposition of the EMI in its two components: Δsaccadic amplitude versus Δcrossover per second. Note: the symbols correspond to the four reader categories defined in Fig. 3(a).

To highlight the effect of a lower signal contrast on the search strategy, we estimated the difference in EMI and the mean number of courses when the signal contrast changed from −50 to −30  HU. To understand the EMI variation, we also estimated the variation (Δ) of its two components when the contrast was decreased: the saccadic amplitude and crossover per second. Figure 9(a) shows ΔEMI versus Δcourse. Figure 9(b) shows Δcrossover per second versus Δsaccadic amplitude for each reader, where Δ is the difference of the considered parameter from −50 to −30  HU. For all readers, Δcourse is positive, whereas for most readers, ΔEMI is negative. This means that when the task becomes more difficult, the EMI tends to decrease and the number of courses to increase. In other words, as the signal contrast lowers, the readers tend to drill more. The fact that Δsaccadic amplitude and Δcrossover per second tend to be negative means that both parameters contribute to the decrease of EMI with decreasing signal contrast.

## 4 Discussion

There is a history of studies investigating how signal contrast and variability in signal shape and size influences visual detection of signal in white noise^10^^,^^17^^,^^18^ and structured anatomical backgrounds.^11^^,^^12^^,^^19^^–^^21^ There is also a long history of studies exploring the types of errors during search with medical images.^8^^,^^14^^–^^16^^,^^22^^–^^25^ Yet most of those studies are restricted to 2-D images.

A number of recent studies have focused on search in 3-D images.^5^^,^^6^^,^^16^^,^^26^^–^^28^ Drew et al.^6^ showed that drillers were superior to scanners along a number of performance metrics, including lung nodule detection rate, percentage of the covered lung parenchyma, and the percentage of search errors. Rubin et al.^5^ reported on radiologists who, while covering only 26% of the lung parenchyma, fixated about 75% of the nodules. Wen et al.^26^ showed that driller fixations were better predicted by dynamical saliency measures than 2-D saliency and thus might explain the higher performance of drillers.

Our study investigated the visual search strategies of radiologists in volumetric images and expanded on current metrics based on eye movement amplitude/crossover to include scrolling behavior. To define the search strategies, we used the previously proposed saccade amplitude/crossover (EMI)^6^ to categorize the readers into drillers and scanners. We first investigated the relationship among the components of the EMI, the saccade amplitude and crossover, and the newly proposed scrolling behavior measure quantified by the number of scrolling direction reversals (number of courses). We found that the correlation between the components of the EMI was much higher than the correlation of the EMI with the number of courses. This finding suggests that the scrolling behavior provides an additional potential source of information about the search strategy of the radiologists. Taking into account, the number of courses to categorize the strategy clearly adds an essential feature in the context of 3-D imaging, because the EMI only quantifies eye movements in the xy plane without allowing for the scrolling in the z direction.

In addition, categorizing radiologists based on the EMI index seemed to vary with signal contrast. Eye movement guidance and strategies during search with 2-D images are known to vary with signal contrast.^29^^,^^30^ For 3-D search, the current study shows that depending on the difficulty of the task, the readers may adopt a strategy, which is a composition of the driller/scanner dichotomy. However, categorizing radiologists based on the number of courses seemed to be more stable across signal contrast.

We also investigated the relationship between the search strategy characterizations (EMI and number of courses) and typical search measures: decision time (mean trial duration), UFOV (mean covered volume), search error rate, and perceptual error rate. We found that the variation in the number of courses across radiologists, unlike the EMI, was significantly related to the decision times and the mean covered volume. Radiologists with a higher number of courses took longer to reach their decision and also explored a larger percentage of the volume. In addition, radiologists with a higher number of courses also resulted in a lower number of trials of missing the lesion and not fixating it (search error rate). This latter result is in line with the results reported by Drew et al.^6^ where a driller’s strategy was characterized as the most effective in studies of volumetric chest image investigations.

Our results also showed that neither the variation in EMI nor in the number of courses was related to variations in the perceptual errors. This is what one might expect since perceptual errors by definition do not involve the search strategy but are rather caused by a failure of perceptual mechanisms at the fovea integrating the visual information to detect or classify the lesion.^31^^,^^32^

Altogether, our findings suggest that coupling the number of courses with the EMI may provide a more complete description of the visual search strategy of radiologists in volumetric images than considering the EMI only. The current results also suggest an expansion of the traditional definitions of scanners and drillers. Scanners, who scroll progressively through the stack of images and focus on different areas within each image slice, could be defined by a high EMI and a low number of courses. Conversely, drillers, who go back and forth through the image stack and tend to focus on a few fixation points, could be defined in terms of a low EMI and a large number of courses.

Using EMI and the number of courses provides us with an explanation of how the strategy evolves when the task becomes more difficult. As shown in Fig. 9(a), lowering the signal contrast from −50 to −30  HU leads to a decrease of EMI and an increased number of courses. In other words, the readers become driller-like when the task is more difficult, with up to 5 additional courses and an EMI that loses up to 0.4 points. Figure 9(b) shows that this decrease of EMI corresponds both to shorter saccades (between 0 and 1 deg shorter) and to fewer crossovers (with a reduction of 0 to 1 crossover per 5 s), which is consistent with lower target detectability in the visual periphery for lower lesion contrast. In other words, the lower the visibility of the lesion in the periphery, the lower the probability that the reader will direct a large saccade toward it.

Our study also confirms what Drew et al.^6^ have shown: drillers are associated with better performance than scanners. This is supported by a significant increase of covered volume with a marginal increase in time, which might enable the reader to reduce search errors. However, because of the correlational nature of the study, we cannot draw causal relationships between the search strategy and error mitigation. The reduction of search errors for radiologists with a higher number of courses could be explained by the larger covered volume, search times but also by some intervening variables such as better ability to detect the lesions in the visual periphery. Establishing a causal relationship between search strategy and performance requires comparing detection rates of the same observers instructed to follow different search strategies.^33^ A recent study with trained observers and simulated images has shown that the impact of search strategy on perceptual performance interacts with the visibility of the signal in the visual periphery.^33^

Correlation between experience and behavior is discussed by Drew et al.^6^ In their study, drillers significantly read more cases per week on average than scanners. In our study, we did not have this information and because we only had one reader with more than 8 years of experience, we did not test for an effect between experience and behavior. That said, we notice that our most experienced readers (17 years) tend to have a scanner strategy for both signal contrasts. Moreover, she has a tendency to be more rapid than the others, with fewer courses and scrolls quickly through the organ of interest.

We identified five main limitations to the current study. The first is associated with classifying each reader into one of four categories through the median split of their EMI or number of courses. This method was adopted to follow a similar analysis by Drew et al.^6^ and allows for a comparison. Yet, the binary categorization has the disadvantage of classifying observers with similar parameters in different categories but on opposite sides of the cutoff threshold. Our results suggest that radiologists lie on a continuous spectrum of scanner and driller strategies rather than only two distinct separate strategies. The second limitation of this study is that we used an identical gaze cone of 5 deg for all readers. In reality, we expect this angle to vary between individual readers.^17^ Furthermore, the signal detectability is known to vary continuously according to the eccentricity,^34^ and not to abruptly become detectable for regions within 5 deg from the fovea.^35^ However, we postulate this should not affect our main observations, which averaged eye movement behavior across 20 readers. The third limitation is related to the demography of our subjects. With only 3 out of 20 radiologists with more than 5 years of professional experience in abdominal cross-sectional imaging, it is possible that their performance might be different from what might be observed in more experienced radiologists. A final limitation arises from the study design. In our experiment, the readers knew that each case had at least one lesion, which is not the case in clinical practice for which a majority of images are lesion free.^36^ The design was chosen to maximize the number of lesion present measurements and missed lesions, which previous studies have shown to be the predominant difference across scanner and driller strategies.^33^ In clinical practice, radiologists are unlikely to explore each case as thoroughly as they did in this study. It is also possible that the driller strategy would not be as efficient in real clinical practice. Future studies should investigate how lesion prevalence interacts with search strategies.^36^^,^^37^

## 5 Conclusion

The current study suggests that the traditional characterization of scanners and drillers might be extended to include the number of scrolled courses as a new component. Our findings can help better characterize search strategies of radiologists reading CT images and further investigate the influence of search strategies on detection performance.
